# Reactive Oxygen Species in the Paraventricular Nucleus of the Hypothalamus Alter Sympathetic Activity During Metabolic Syndrome

**DOI:** 10.3389/fphys.2015.00384

**Published:** 2015-12-23

**Authors:** Josiane C. Cruz, Atalia F. L. Flôr, Maria S. França-Silva, Camille M. Balarini, Valdir A. Braga

**Affiliations:** ^1^Centro de Biotecnologia, Universidade Federal da ParaíbaJoão Pessoa, Brazil; ^2^Centro de Ciências da Saúde, Universidade Federal da ParaíbaJoão Pessoa, Brazil

**Keywords:** paraventricular nucleus of the hypothalamus, angiotensin II, oxidative stress, metabolic syndrome

## Abstract

The paraventricular nucleus of the hypothalamus (PVN) contains heterogeneous populations of neurons involved in autonomic and neuroendocrine regulation. The PVN plays an important role in the sympathoexcitatory response to increasing circulating levels of angiotensin II (Ang-II), which activates AT1 receptors in the circumventricular organs (OCVs), mainly in the subfornical organ (SFO). Circulating Ang-II induces a *de novo* synthesis of Ang-II in SFO neurons projecting to pre-autonomic PVN neurons. Activation of AT1 receptors induces intracellular increases in reactive oxygen species (ROS), leading to increases in sympathetic nerve activity (SNA). Chronic sympathetic nerve activation promotes a series of metabolic disorders that characterizes the metabolic syndrome (MetS): dyslipidemia, hyperinsulinemia, glucose intolerance, hyperleptinemia and elevated plasma hormone levels, such as noradrenaline, glucocorticoids, leptin, insulin, and Ang-II. This review will discuss the contribution of our laboratory and others regarding the sympathoexcitation caused by peripheral Ang-II-induced reactive oxygen species along the subfornical organ and paraventricular nucleus of the hypothalamus. We hypothesize that this mechanism could be involved in metabolic disorders underlying MetS.

## Introduction

The sympathoexcitation is linked to various diseases; circulating levels of Ang-II modulate SFO angiotensinergic projection to pre-autonomic PVN neurons, which lead to increases in sympathoexcitatory activity to the spinal cord via direct projections (Koshiya and Guyenet, 1996; Badoer, 2001; Stocker et al., 2004) or indirectly projecting to pre-sympathetic neurons in the RVLM (Koshiya and Guyenet, 1996; Badoer, 2001; Ito et al., 2002; Stocker et al., 2004). Increasing evidence supports the premise that Ang-II in the PVN is involved in pathological conditions originating from sympathoexcitation, such as hypertension, heart failure, diabetes, obesity, and metabolic syndrome (Gutkind et al., 1988; Ito et al., 2002; Oliveira-Sales et al., 2009; Braga et al., 2011). It has also been demonstrated that Ang-II increase reactive oxygen species (ROS) along the subfornical organ–paraventricular nucleus of the hypothalamus–rostral ventrolateral medulla axis [SFO-PVN-RVLM axis (Oliveira-Sales et al., 2008; Braga et al., 2011; Burmeister et al., 2011)].

The main ROS within the central nervous system is superoxide anions (O2−). Within the PVN, superoxide accumulation within the PVN ultimately results in sympathetic overactivity (Oliveira-Sales et al., 2009; Peterson et al., 2009; Burmeister et al., 2011; Cardinale et al., 2012; Campos et al., 2015). The aim of this mini-review is to discuss the role of sympathoexcitation induced by Ang-II-dependent ROS production in the pre-autonomic PVN neurons in modulating the development and/or metabolic disorders that results in the development and/or the maintenance of metabolic syndrome.

### Anatomical and functional organization of PVN

The paraventricular nucleus of the hypothalamus is anatomically connected to other hypothalamic areas and to the brainstem, playing a pivotal role in several homeostatic responses, being an important integrative nucleus for autonomic and neuroendocrine functions (Swanson and Sawchenko, 1980; Stern, 2001; Cruz et al., 2008; Cruz and Machado, 2009; Reis et al., 2010). Among the PVN functions are regulation of food intake, adipose afferent reflex (AAR), responses to stress, modulation of metabolic rate, thermoregulation, modulation of sympathetic nerve activity, and cardiovascular function (Swanson and Sawchenko, 1980; Stern, 2001; Benarroch, 2005; Cruz et al., 2008; Cruz and Machado, 2009; Reis et al., 2010; Cassaglia et al., 2011; Zsombok et al., 2011; Zhang et al., 2012; Ding et al., 2013; Xiong et al., 2014). The PVN is comprised of magnocellular and parvocellular subnuclei, which have different properties both neurochemically and electrophysiologically (Swanson and Sawchenko, 1980; Stern, 2001). The magnocellular subnucleus projects to the posterior hypophysis and parvocellular subnucleus, which include pre-autonomic neurons, send descending projections to cardiovascular autonomic brainstem nuclei as well as direct projections to the spinal cord (Koshiya and Guyenet, 1996; Badoer, 2001; Stocker et al., 2004; Cruz et al., 2008). Therefore, electrophysiological and functional studies support an essential role for the PVN in central blood pressure control (Cruz and Machado, 2009; Cruz et al., 2010; Busnardo et al., 2013) and sympathetic nerve activity (Koshiya and Guyenet, 1996; Badoer, 2001; Stocker et al., 2004). Our previous studies suggest that parvocellular pre-autonomic neurons modulate baseline blood pressure through activation of glutamatergic, GABAergic, purinergic, nitrergic, and angiotensinergic mechanisms (Chen et al., 2003; Cruz and Machado, 2009; Cruz et al., 2010; Busnardo et al., 2013). Accumulating evidence support the idea that imbalance between paraventricular inhibitory GABAergic and excitatory glutamatergic and/or angiotensinergic neurotransmission in the PVN, contribute to increase the pre-autonomic neuronal drive mediating neurogenic hypertension (Gören et al., 2000; Chen et al., 2003; Li and Pan, 2005; Li et al., 2006; Oliveira-Sales et al., 2009). Magnocellular and parvocellular neurons from PVN express receptors to a wide range of neurotransmitters and neurohormones including leptin, insulin, neuropeptide Y, Ang-II, GABA, glutamate, vasopressin, oxytocin, and noradrenaline (Stanley and Leibowitz, 1984; Saphier and Feldman, 1991; Lenkei et al., 1997; Håkansson and Meister, 1998; Zsombok et al., 2011). Therefore, it is suggested that an imbalance in synaptic function that modulates the pre-autonomic or neurosecretory neuron results in cardiovascular and neuroendocrine dysfunctions that, in turn, contribute to the development and potentiation of sympathoexcitatory response observed in hypertension, heart failure, atherosclerosis, diabetes, and obesity.

### SFO-PVN-RVLM pathway for circulating Ang-II is involved in the cardiovascular regulation

Some circulating lipophobic substances, incapable of crossing the blood brain barrier (BBB), such as glucose, insulin, leptin, noradrenaline, and angiotensin II have their receptors expressed in neurons of the circunventricular organs (CVOs), which have an incomplete BBB (Lenkei et al., 1997; Boundy and Cincotta, 2000; Braga et al., 2011; Cassaglia et al., 2011; Lob et al., 2013; Prior et al., 2014). One of the major CVOs receiving information from the peripheral circulation is the subfornical organ (SFO). Anatomical and functional evidence suggest that SFO is a pivotal nucleus in modulating pressor and dipsogenic actions of circulating Ang II (Bains et al., 1992; Li and Ferguson, 1993; Sakai et al., 2007; Braga et al., 2011). Genetic and physiological evidence shows that circulating Ang-II is involved in *de novo* synthesis of Ang II within the SFO, which is an integrative mechanism of fluid and cardiovascular homeostasis (Bains et al., 1992; Li and Ferguson, 1993; Sakai et al., 2007; Burmeister et al., 2011). Angiotensin II AT1 and AT2 receptors (AT1R; AT2R) are expressed in neurons and astrocytes of the PVN (Lenkei et al., 1997; Coleman et al., 2009; Oliveira-Sales et al., 2009) and angiotensinergic connections between the SFO and PVN is describe to control drinking and sympathetic nerve activity (Gutkind et al., 1988; Bains et al., 1992; Li and Ferguson, 1993; Anderson et al., 2001; Sakai et al., 2007; Burmeister et al., 2011). In that regard, several studies show that angiotensinergic connections between SFO and PVN are involved in the generation and maintenance of elevated baseline blood pressure in hypertensive rats (Gutkind et al., 1988; Burmeister et al., 2011). For example, studies by Miyakubo et al. (2002) demonstrated that excitatory response elicited by Ang-II in SFO neurons projecting to PVN was higher in spontaneous hypertensive rats (SHR) than in normotensive Wistar Kyoto rats (WKY). The brain Ang-II neurocircuitary also involves pre-autonomic PVN neurons projecting to rostral ventrolateral medulla (RVLM). The RVLM which tonically controls sympathetic vasomotor activity (Guyenet et al., 1989). Studies by Ito et al. (2002) indicate that RVLM vasomotor neurons in SHR, but not in the WKY rats, are tonically excited by PVN driven angiotensin II projections. Furthermore, several studies support the idea that Ang-II along the SFO-PVN-RVLM axis is a significant neuronal pathway involved in the maintenance of neurogenic hypertension (Ito et al., 2002; Oliveira-Sales et al., 2009; Braga et al., 2011).

### Ang-II induced ROS accumulation along the SFO-PVN-RVLM axis contributing to the pathogenesis of hypertension

Accumulating evidence support the idea, that Ang-II-induced oxidative stress within the PVN contributes to the pathogenesis of hypertension. In addition to increasing blood pressure and sympathetic nerve activity, central infusion of Ang-II leads to elevated levels of neurotransmitters (glutamate and norepinephrine), AT1R, pro-inflammatory cytokines, phosphorylated IKKbeta, NF-kappaB subunits, and superoxide in the central nervous system (Erdös et al., 2006; Oliveira-Sales et al., 2009; Peterson et al., 2009; Burmeister et al., 2011).

There is now growing evidence suggesting that inflammation and central Ang-II-induced ROS production are involved in the pathogenesis of neurogenic hypertension. For example, in Ang-II-treated rats, bilateral microinjection of NFkappaB blocker into the PVN induces a local decrease in NFkappaB p65 subunit activity, proinflammatory cytokines, ROS, AT1-R, as well as in blood pressure (Cardinale et al., 2012).

Ang-II acting on the AT1R induces activation of NADPH oxidase through protein kinase C (PKC). The NADPH oxidase is the major source of superoxide anion. This enzyme is composed of catalytic membrane (gp91^phox^ and p22^phox^) and cytoplasmic (p40^phox^, p47^phox^, and p67^phox^) subunits, which transfer electrons to molecular oxygen, producing reactive oxygen species as superoxide (Chabrashvili et al., 2002; Lassègue and Clempus, 2003).

Oxidative stress is characterized by an imbalance between the production of ROS and antioxidant systems (Betteridge, 2000). Numerous studies support the concept that ROS production is increased in different nuclei in the brainstem and hypothalamus (Oliveira-Sales et al., 2009; Peterson et al., 2009; Braga et al., 2011; Burmeister et al., 2011; Campos et al., 2015). The role of oxidative stress in the development and/or maintenance of neurogenic hypertension has recently been reported in several animal models of hypertension, including the renovascular two-kidney–one-clip model [2K1C (Oliveira-Sales et al., 2008, 2009; Burmeister et al., 2011; de Queiroz et al., 2013)]. Studies by Lob et al. (2013) showed an increase in the superoxide production in the SFO after chronic angiotensin II infusion, which was blunted by SFO-targeted injections of an adenovirus encoding cre-recombinase for reducing of p22 (phox), Nox2, and Nox4 mRNA expression. In addition, studies by Yuan et al. (2013) showed that superoxide dismutase 1 (SOD1), an antioxidant enzyme, was overexpressed in the PVN, attenuating augmented sympathetic activity, and cardiac sympathetic afferent reflex, while improving the myocardial and vascular remodeling in spontaneous hypertensive rats (SHR). The expression of the isoforms Nox1, Nox2, and predominant Nox4 mRNA were found in the PVN under basal conditions. Furthermore, Nox4-generated superoxide within the PVN contributes to the sympathoexcitation and cardiac dysfunction observed in mice that experienced heart failure (Infanger et al., 2010).

It has been suggested that upregulation of ROS in the RVLM and PVN contributes to increased blood pressure and SNA in renovascular hypertensive rats, with ROS preceding the increase in blood pressure in Ang-II-dependent model of hypertension (Kitiyakara and Wilcox, 1998; Botelho-Ono et al., 2011; Burmeister et al., 2011; de Queiroz et al., 2013) mRNA expression studies revealed that AT-1 and NADPH oxidase subunits were greater in the RVLM and PVN in renovascular hypertensive rats (Oliveira-Sales et al., 2009; Campos et al., 2015). In addition, studies by Burmeister et al. (2011) documented that a significant increase in the superoxide production in the PVN of renovascular hypertensive mice leads to activator protein-1 (AP-1) activation, a nuclear transcription factor, resulting in hypertension, while inhibition of AP-1 activity in the prevented renovascular hypertension. Furthermore, microinjection of superoxide dismutase mimetic, 4 hydroxy-2, 2, 6, 6-tetramethyl piperidinoxyl (Tempol) into the RVLM and PVN decreased the mean arterial pressure and renal sympathetic nerve activity in renovascular hypertensive rats, supporting the idea that upregulation of ROS in central cardiovascular areas, such as RVLM and PVN, contributes to elevated arterial pressure and sympathetic activity (Oliveira-Sales et al., 2009). Interestingly, microinjection of an adenovirus (Ad) encoding superoxide dismutase (AdCuZnSOD) in the PVN not only decreased the local superoxide accumulation into the PVN, but also prevented hypertension. Together, these observations led to the proposal (Braga et al., 2011) that the formation of Ang-II-induced ROS along the SFO-PVN-RVLM axis is an important mechanism involved in the pathogenesis of neurogenic hypertension.

### Ang-II, obesity and diabetes cross-talk in the PVN

Diet and lifestyle associated to genetic factors are involved in the development of metabolic syndrome (MetS). Metabolic changes such as dyslipidemia, glucose intolerance, hyperinsulinemia, hyperleptinemia, systemic inflammation, and chronic increase in the SNA, which characterize MetS, also augment the risk of developing diseases such as obesity, diabetes, atherosclerosis, and arterial hypertension. The PVN, as described above, is a key central nucleus participating in the regulation of cardiovascular and sympathetic activity (Koshiya and Guyenet, 1996; Badoer, 2001; Stocker et al., 2004; Cruz et al., 2008). It is involved in the sympathetic overactivity in rats with hypertension (Gören et al., 2000; Chen et al., 2003; Li et al., 2006; Oliveira-Sales et al., 2009), obesity (Xiong et al., 2012; Ding et al., 2013), and insulin resistance [commonly seen in the form of diabetes (Cassaglia et al., 2011; Zhang et al., 2012)]. In addition, several reports suggest that ROS activation contributes to insulin resistance observed in diabetic rats accompanied by obesity and hypertension (Folli et al., 1997; Xiong et al., 2012; Zhang et al., 2012; Cruz et al., 2013; Ding et al., 2013; de Kloet et al., 2013)

It is known that activation of the renin-angiotensin system may lead to insulin resistance in the vasculature (Folli et al., 1997); Ang-II impairs insulin receptor intracellular signaling, inhibiting insulin receptor substrate-1 (IRS-1) phosphorylation and phosphatidylinositol (PI) 3–kinase activation (Folli et al., 1997; Cizmeci and Arkun, 2013). In addition, it has been documented that AT1R receptor expression is increased in the PVN of rats with diabetes and insulin resistance (Zhang et al., 2012; Sun et al., 2014). Furthermore, Ang-II activates NADPH oxidase via AT1 receptors, increasing superoxide anion accumulation in the PVN, thereby contributing to enhanced sympathetic activity in diabetic and insulin resistance rats (Patel et al., 2011; Zhang et al., 2012; Sun et al., 2014).

Sympathetically-mediated interactions between PVN and white adipose tissue via AAR are important for the maintenance of total body fat and energy balance (Xiong et al., 2012; Ding et al., 2013). AAR is increased in obese hypertensive rats (Xiong et al., 2012, 2014; Ding et al., 2013) and inhibition of PVN decreases SNA and mean arterial pressure, while abolishing AAR in hypertensive obese rats (Xiong et al., 2012). Furthermore, studies by Ding et al. (2013) showed that NADPH oxidase-derived superoxide anions in the PVN modulates AAR, while PVN microinjection of tempol decreases baseline renal SNA, blood pressure, and attenuated the AAR. Thus, Ang-II induces ROS in the PVN may be a significant central mechanism modulating AAR. Studies by de Kloet et al. (2013) observed that deletion of AT1 receptors in the PVN not only reduced the local expression of corticotrophin-releasing hormone (CRH), oxytocin, and tumor necrosis factor α (TNF-α, a pro-inflammatory cytokine), but also decreased systolic blood pressure in mice rendered obese by high fat diet. This suggests that AT1 receptors in the PVN regulates the central metabolic changes that promotes metabolic and cardiovascular disorders.

## Conclusion

**Figure 1:**
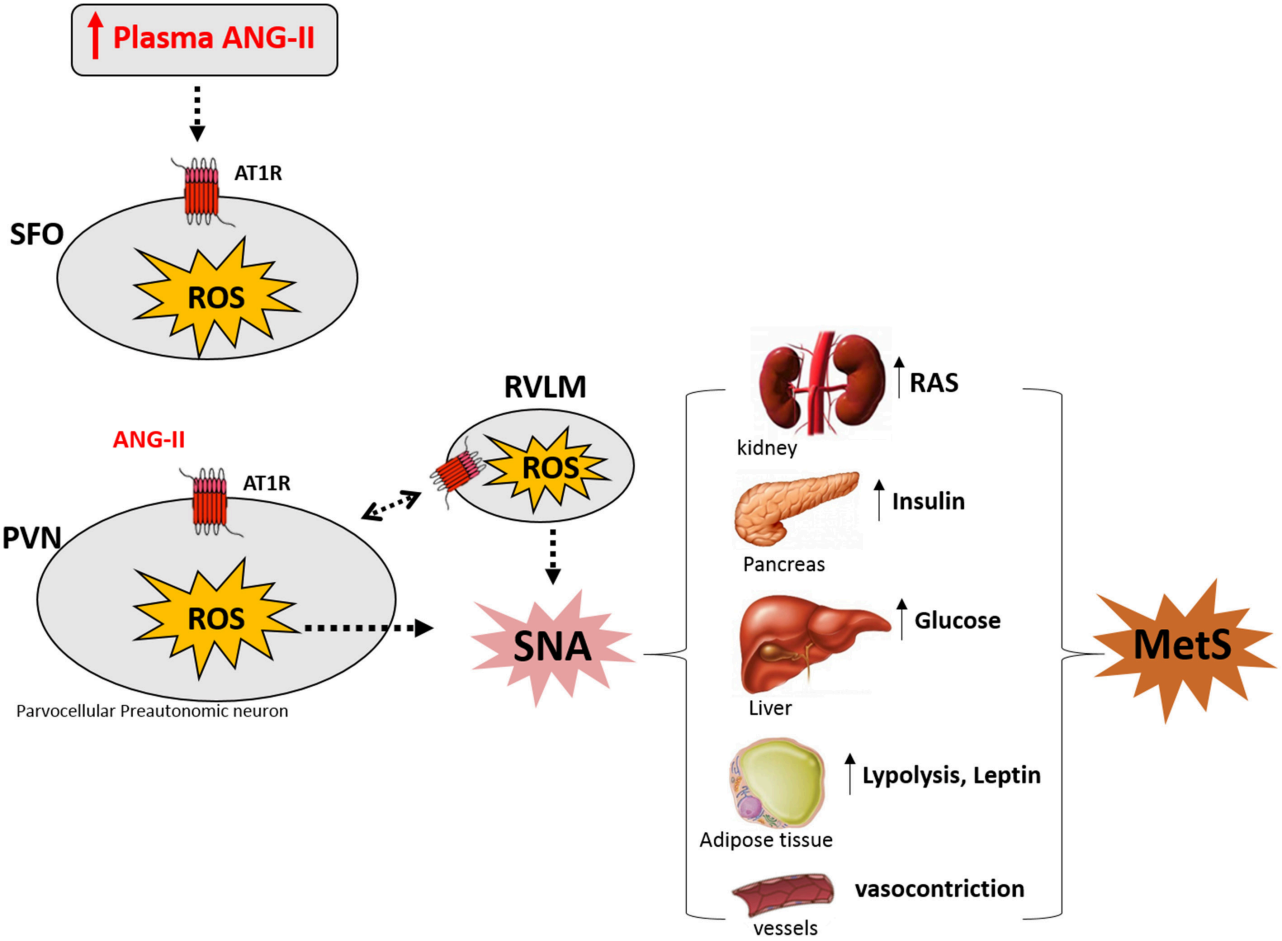
**Angiotensin II (Ang-II) induces sympathetic nerve activation (SNA) by increasing reactive oxygen species (ROS) along SFO-PVN axis underlying Metabolic Syndrome (MetS) symptoms (see text)**. Modified by de Queiroz et al. (2013) and Benarroch (2005). SFO: subfonical organ; PVN, paraventricular nucleus of the hypothalamus; RVLM, rostral ventrolateral medulla; AT1R, AT1 Ang-II receptor; RAS, renin angiotensin system.

In the last years, our laboratory has been investigating the mechanisms underlying neurogenic hypertension and our results strongly suggest that this pathological condition is caused by Ang-II-dependent ROS accumulation along the SFO-PVN-RVLM axis (Peterson et al., 2009; Botelho-Ono et al., 2011; Braga et al., 2011; Burmeister et al., 2011; de Queiroz et al., 2013). Accumulating evidence suggest that hyperactivity of the angiotensin system within the PVN is involved not only in hypertension, but also in diabetes and obesity existing as comorbidities (Oliveira-Sales et al., 2008, 2009; de Kloet et al., 2010, 2013; Braga et al., 2011; Xiong et al., 2012, 2014; Cizmeci and Arkun, 2013; Ding et al., 2013). This mini-review supports the hypothesis illustrated in the Figure 1 that the increase in the circulating levels of Ang-II activates angiotensinergic neurons in the SFO, which projects to pre-autonomic neurons expressing AT1 receptors in the PVN. The stimulation of AT1 receptors in the PVN and RVLM induces intracellular signals activating NADPH oxidase through protein kinase C. NADPH oxidase activity increases ROS formation, contributing to overactivity of pre-autonomic PVN neurons, resulting in sympathoexcitation through an indirect pathway (angiotensinergic projections to RVLM) and/or directly projections to the spinal cord, thereby mediating increase in plasma renin-angiotensin system, insulin, glucose, leptin, lipolysis as well as vasoconstriction. All these metabolic changes are involved in the symptoms of MetS.

## Author contributions

All authors participated in the design of the manuscript, drafted the manuscript, revised the manuscript critically and approved the final version.

### Conflict of interest statement

The authors declare that the research was conducted in the absence of any commercial or financial relationships that could be construed as a potential conflict of interest.
